# FTIR, Weight, and Surface Morphology of Poly(vinyl chloride) Doped with Tin Complexes Containing Aromatic and Heterocyclic Moieties

**DOI:** 10.3390/polym13193264

**Published:** 2021-09-25

**Authors:** Anaheed A. Yaseen, Emad Yousif, Emaad T. B. Al-Tikrity, Gamal A. El-Hiti, Benson M. Kariuki, Dina S. Ahmed, Muna Bufaroosha

**Affiliations:** 1 Department of Chemistry, College of Science, Tikrit University, Tikrit 34001, Iraq; ch@sc.nahrainuniv.edu.iq (A.A.Y.); emaad1954@tu.edu.iq (E.T.B.A.-T.); 2 Department of Chemistry, College of Science, Al-Nahrain University, Baghdad 64021, Iraq; emad.yousif@nahrainuniv.edu.iq; 3 Department of Optometry, College of Applied Medical Sciences, King Saud University, P.O. Box 10219, Riyadh 11433, Saudi Arabia; 4 School of Chemistry, Cardiff University, Main Building, Park Place, Cardiff CF10 3AT, UK; kariukib@cardiff.ac.uk; 5 Department of Medical Instrumentation Engineering, Al-Mansour University College, Baghdad 64021, Iraq; dina.saadi@muc.edu.iq; 6 Department of Chemistry, College of Science, United Arab Emirates University, P.O. Box 15551, Al-Ain 1818, United Arab Emirates; muna.bufaroosha@uaeu.ac.ae

**Keywords:** trimethoprim–tin complexes, poly(vinyl chloride), roughness factor, photodegradation, photo-oxidation, weight loss, functional group index

## Abstract

Poly(vinyl chloride) (PVC) is an important synthetic plastic that is produced in large quantities (millions of tons) annually. Additives to PVC are necessary to allow its use in many applications, particularly in harsh conditions. In regard to this, investigation of the synthesis of trimethoprim–tin complexes and their use as PVC additives is reported. Trimethoprim–tin complexes were obtained from the reaction of trimethoprim and tin chlorides using simple procedures. Trimethoprim–tin complexes (0.5% by weight) were added to PVC to produce homogenous mixtures and thin films were made. The effect of ultraviolet irradiation on the surface and properties of the PVC films was investigated. The level of both photodecomposition and photo-oxidation of PVC films containing trimethoprim–tin complexes was observed to be lower than for the blank film. The effectiveness of tin complexes as PVC photostabilizers reflects the aromaticity of the additives. The complex containing three phenyl groups attached to the tin cation showed the most stabilizing effect on PVC. The complex containing two phenyl groups was next, with the one containing butyl substituents resulting in the least stabilization of PVC. A number of mechanisms have been proposed to explain the role of the synthesized complexes in PVC photostabilization.

## 1. Introduction

Plastics are synthetic polymers that can be molded into different shapes. Plastics are cheap, durable, lightweight, and can be used in a myriad of applications. They are effective replacements for glass, wood, and steel [1,2,3]. Plastics are mainly produced from fossil feedstocks relying on technology that was developed a long time ago. Physical properties of plastics such as strength, color, density, molecular weight, and rigidity can be manipulated during the manufacturing processes. Polyethylene, polypropylene, poly(vinyl chloride) (PVC), polyethylene terephthalate, and polystyrene are the most common synthetic plastics. In Europe, the plastics industry contributed to 1.5 million jobs and generated EUR 350 billion in 2019 [4]. China is the main producer of plastics and contributes 51% of the world’s plastics production. Plastics are mainly used in packaging (39.6%), construction materials (20.4%), automotive (9.6%), electronics (6.2%), household (4.1%), agriculture (3.4%), and other (16.7%) applications [4]. Large quantities (4 million tons) of plastics were recycled and reused in Europe in 2018. However, the recycling process is tedious, energy-, money-, and time-consuming, and involves many steps, namely collection, first sorting, shredding, washing, second sorting, and extrusion [4].

PVC is an important thermoplastic with many advantages. PVC has a low price, high strength and stability, resists attack by chemicals and corrosion in harsh environments, and acts as an electrical insulator, smoke suppressor, and flame retardant due to its high content of chlorine (approximately 57% by weight) [5]. These properties make PVC suitable for a broad range of applications [6]. The production scale for PVC has increased over the years and this trend will undoubtedly continue into the future [7]. The versatility of PVC can be increased by manipulation of its flexibility to enable its use in applications that require soft polymers via the addition of plasticizers [8]. Plasticizers create free volume by being located between the PVC chains. Another mechanism by which plasticizers soften PVC is through lowering the glass transition temperature [9,10].

A disadvantage of PVC is that it suffers from photodegradation at temperatures above 100 °C and in the process releases hydrogen chloride (HCl) forming an unstable structure. The elimination of HCl leads to the formation of polyenes containing conjugated carbon–carbon double bonds. The dehydrochlorination process results in deterioration of physical and thermal properties of PVC along with discoloration and therefore limits its outdoor applications [11,12]. Although PVC does not absorb high wavelength UV light (λ > 220 nm), polyene chains produced as a result of defects can initiate photodegradation of the polymer [13,14]. It is therefore necessary to use PVC photostabilizers in order to limit the damage caused by photoirradiation [15]. Consequently, there is a continuing need to improve as well as produce new PVC additives.

PVC additives act mainly as quenchers for radicals, peroxides, heat, and UV energy [16,17]. The main role of PVC stabilizers is inhibition of the formation of allylic chlorides and HCl [18,19]. The additives should be used in minimal quantity to avoid color change, be blended well with the polymer, be cheap to produce and involatile. In addition, they should not leak or pose a danger to the environment. Many potential additives are banned from use due to their toxicity and the harm they cause to humans and animals. Banned additives include flame retardants (e.g., chlorinated biphenyls), plasticizers (e.g., phthalates), stabilizers (e.g., phosphites), and those containing metals [20,21,22,23]. Various additives such as polyphosphates [24], polybenzimidazoles [25], metal oxides [26,27,28], pigments [29], Schiff bases [30,31,32,33,34], and substituted tin complexes containing aromatic moieties [35,36,37,38] have been used to improve photostability of PVC.

The current work reports the successful synthesis and use of trimethoprim–tin complexes as PVC additives. Trimethoprim inhibits bacterial growth and is used to treat cystitis, acne, and urinary tract and chest infections [39]. It is a colorless solid, commercially available, contains pyrimidine and aryl rings (aromatic moieties), and heteroatoms (nitrogen and oxygen). It can coordinate with the PVC, does not alter the color, absorbs the UV light, and quenches radicals and peroxides. In addition, the tin cation acts as a quencher for the HCl produced during PVC photodegradation. Therefore, trimethoprim–tin complexes have all the qualities required in a photostabilizer. It is also anticipated that the inclusion of extra aromatic substituents (phenyl groups) on the skeleton of the tin complexes can enhance their ability to photostabilize PVC.

## 2. Materials and Methods

### 2.1. General

Trimethoprim (98%), triphenyltin chloride (Ph_3_SnCl; 95%), diphenyltin dichloride (Ph_2_SnCl_2_; 96%), di-*n*-butyltin dichloride (*n*-Bu_2_SnCl_2_; 96%), and solvents were purchased from Merck (Gillingham, UK). PVC with an average molecular weight of approximately 233,000 and a polymerization degree of 800 was supplied by Petkim Petrokimya (Istanbul, Turkey). The amounts of carbon, hydrogen, nitrogen, and tin within the complexes were determined using a Vario EL III elemental analyzer (Elementar Americas, Ronkonkoma, NY, USA). An FTIR 8400 Shimadzu spectrophotometer (Shimadzu, Tokyo, Japan) was used to record the FTIR spectra. A Bruker DRX-500 NMR spectrometer (Zürich, Switzerland) was used to record the ^1^H and ^13^C NMR at 500 and 125 MHz, respectively. The NMR spectra were recorded in deuterated dimethyl sulfoxide at 25 °C. The chemical shifts (ppm) were measured relative to tetramethylsilane as a standard. An accelerated weather-meter QUV tester (Q-Panel Company; Homestead, FL, USA) was used at 25 °C for the UV irradiation of films. The optical images of the surface of samples were captured using a Meiji Techno microscope (Tokyo, Japan). The field emission scanning electron microscopy (FESEM) images were obtained using a SIGMA 500 VP microscope (ZEISS Microscopy, Jena, Germany) in which the accelerating voltage was 15 kV at 25 °C. A Hitachi H-7100 electron microscope (London, UK) was used to record the transmission electron micrograph (TEM) images and a Veeco instrument (Plainview, NY, USA) was used to capture the atomic force microscopy (AFM) images.

### 2.2. Synthesis of Complex ***1***

**Scheme 1:**
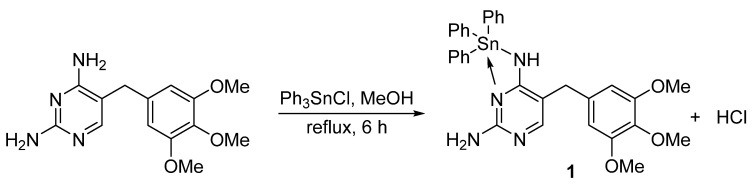
Synthesis of trimethoprim–tin complex **1**.

A solution of Ph_3_SnCl (0.39 g, 1.0 mmol) in methanol (MeOH; 5 mL) was added to a stirred solution of trimethoprim (0.29 g, 1.0 mmol) in MeOH (10 mL). This mixture was heated under reflux for 6 h. The solid produced on cooling was filtered, washed with MeOH (2 × 5 mL), and dried to give **1** (Scheme 1) in 75% yield (Table 1).

### 2.3. Synthesis of Complexes ***2*** and ***3***

**Scheme 2:**
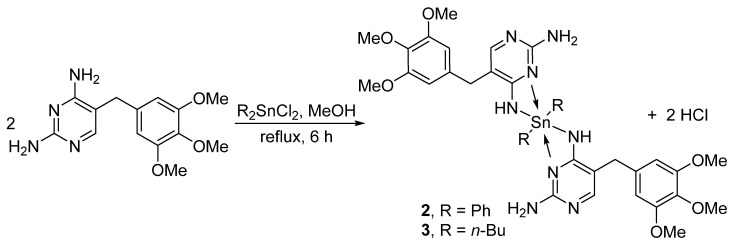
Synthesis of trimethoprim–tin complexes **2** and **3**.

A solution of Ph_2_SnCl_2_ (0.35 g, 1.0 mmol) or *n*-Bu_2_SnCl_2_ (0.30 g, 1.0 mmol) in MeOH (5 mL) was added to a stirred solution of trimethoprim (0.58 g, 2.0 mmol) in MeOH (10 mL). The mixture was heated under reflux for 6 h. The solid produced on cooling was filtered, washed with MeOH (2 × 5 mL), and dried to give **2** or **3** (Scheme 2) in 79 or 82% yield, respectively (Table 1).

### 2.4. Preparation of PVC Films

Complex **1**, **2**, or **3** (25 mg) was added to a magnetically stirred suspension of PVC (5.0 g) in tetrahydrofuran (THF; 100 mL) and the mixture was stirred for 2 h. The homogenous solution obtained was poured onto a clean and dry glass plate (4 × 4 cm^2^) containing 15 holes with a thickness of approximately 40 µ. The solvent was removed by leaving the plate at 25 °C for 18 h. The films produced were dried in a vacuum oven (25 °C) for 18 h.

### 2.5. Irradiation of PVC Films

A UV light (λ_max_ = 365 nm; light intensity = 6.2 × 10^−9^ ein dm^−3^ s ^−1^) was used for the irradiation of the PVC samples. The sample was rotated regularly to ensure that it was exposed to the light evenly from all sides. The films were irradiated for a time that ranged from 0 to 300 h. The effect the irradiation on the PVC properties was recorded every 50 h.

### 2.6. Measurement of PVC Functional Group Indices

PVC photodegradation leads to the formation of residues that contain C=O (carbonyl) and C=C (unsaturated carbons) groups. The growth of the intensity of the bands for both C=O and C=C groups generated by photoirradiation can be monitored using FTIR spectroscopy and compared with that for the C–H bond as a reference [40]. The indices of C=O and C=C groups (*Is*) were calculated using Equation (1), where *A_s_* and *A_r_* are the absorbance of the functional and reference groups, respectively.

*I_S_* = *A_S_*/*A_r_* (1)

### 2.7. Measurement of PVC Weight Loss

PVC photodegradation leads to weight loss due to the elimination of volatile molecules. The weight loss (%) can be calculated from the weight of the sample pre- (*W_pre_*) and post-irradiation (*W_post_*) using Equation (2) [41].

Weight loss (%) = (*W_pre_* − *W_post_*)/*W_pre_* × 100 (2)

## 3. Results and Discussion

### 3.1. Synthesis of Trimethoprim–tin Complexes ***1–3***

The reaction of trimethoprim and Ph_3_SnCl in a 1:1 molar ratio in MeOH under reflux gave complex **1** (Scheme 1) in 75% yield (Table 1). Similarly, the reaction of trimethoprim and Ph_2_SnCl_2_ or *n*-Bu_2_SnCl_2_ in a 2:1 molar ratio under similar reaction conditions gave **2** or **3** (Scheme 2) in 79 or 82% yield (Table 1). The purity of the synthesized complexes was confirmed by elemental analyses (Table 1).

The absorption bands due to vibration of the NH_2_, NH, and C=N groups appeared in the 3406–3408 cm^−1^, 3163–3180 cm^−1^, and 1680–1682 cm^−1^ regions, respectively (Table 2) in the FTIR spectra of **1–3** (Figures S1–S3). In addition, the bands that appeared in the 605–507 cm^−1^ and 497–586 cm^−1^ regions are attributed to the vibration of the Sn–C, and Sn–N groups, respectively.

The ^1^H NMR spectra of **1–3** indicated the presence of the pyrimidine proton that appeared as a singlet at low magnetic field. The NH proton appeared as an exchangeable singlet at high magnetic field due to the shielding effect of the Sn atom. The spectra showed all other protons (Table 3). The ^13^C NMR spectra of trimethoprim–tin complexes showed the presence of carbons derived from both the trimethoprim unit and substituents (phenyl and butyl groups) attached to the Sn atom (Table 3). Representative NMR spectra are shown in Figures S4 and S5.

### 3.2. Investigation of PVC Photodegradation through FTIR Spectrophotometry

**Figure 1:**
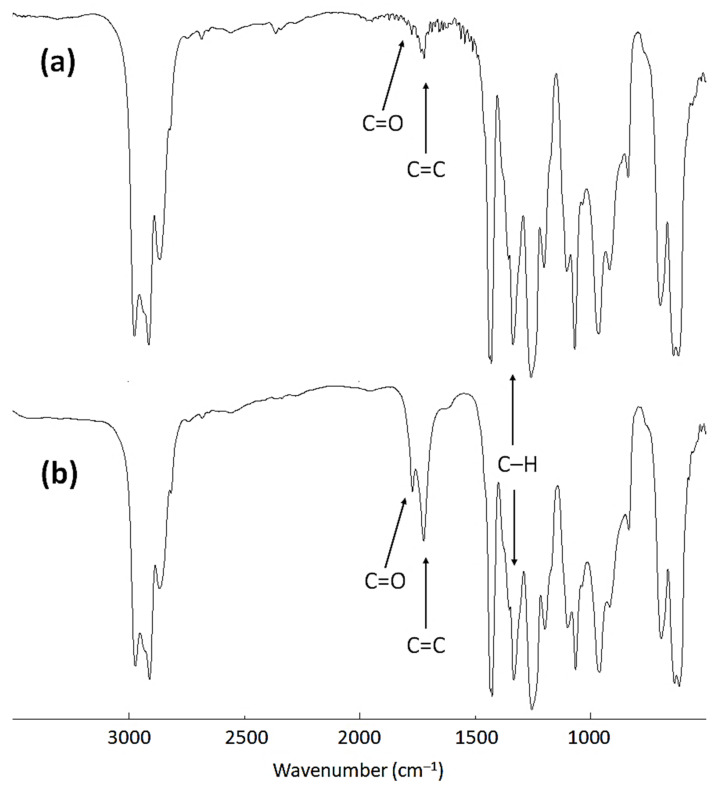
FTIR spectra of the pure PVC film pre- and post-irradiation.

PVC photodegradation takes place mainly due to the elimination of free radicals (e.g., Cl^.^) and the release of HCl. This process of dehydrochlorination is responsible for the formation of short polymeric chains containing C=O and –CH=CH– moieties [42,43,44]. As irradiation of PVC progresses, the formation of the C=O and –CH=CH– becomes noticeably significant. Therefore, FTIR spectroscopy was used to examine the growth of the absorption bands corresponding to the C=O (1722 cm^−1^) and –CH=CH– (1602 cm^−1^) groups during the irradiation process (Figure 1). The intensities of these absorption bands increased substantially during irradiation compared with that for the C–H bonds (CH_2_; 1328 cm^−1^) of the PVC chains. The UV light does not affect the intensity of the absorption band corresponding to the reference (C–H bonds).

**Figure 2:**
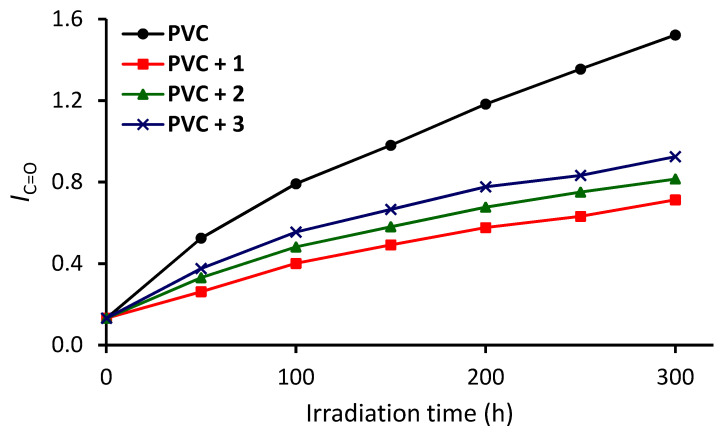
Changes in the *I_C=O_* index of PVC films.

**Figure 3:**
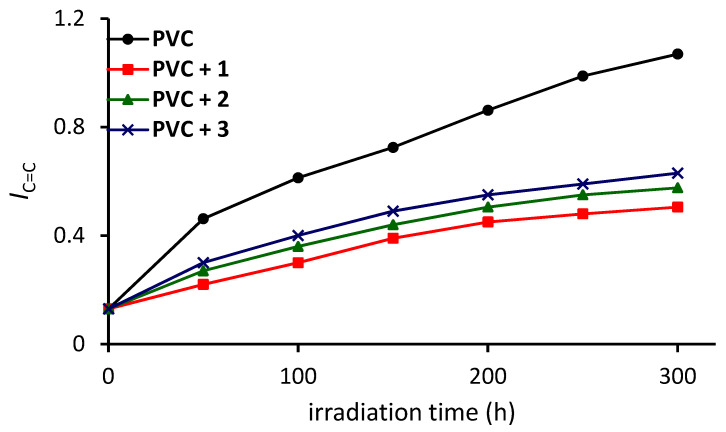
Changes in the *I_C=C_* index of PVC films.

Equation (1) has been used to calculate the indices of both C=O (*I_C=O_*) and C=C (*I_C=C_*) groups for the pure PVC film and the blends containing **1–3** after every 50 h of irradiation. The changes in *I_C=O_* and *I_C=C_* for the PVC during irradiation are shown in Figure 2 and Figure 3, respectively. It was clear that the changes in the *I_C=O_* and *I_C=C_* were significantly higher and sharper in the case of the pure PVC film. Complexes **1–3** evidently reduced the formation of small fragments containing C=O and C=C groups and hence acted as PVC photostabilizers. For the pure PVC film, the *I_C=C_* was 0.53 after 50 h, 0.80 after 100 h, 1.01 after 200 h, and 1.51 after 300 h of irradiation. The PVC film containing **1** showed the lowest *I_C=O_* at 0.26 after 50 h, 0.40 after 100 h, 0.58 after 200 h, and 0.71 after 300 h of irradiation. The superior ability of complex **1** to act as a UV absorber is ascribed to the three phenyl group substituents. A similar pattern is observed for the changes in *I_C=C_*.

### 3.3. Investigation of PVC Photodegradation through Weight Loss

**Figure 4:**
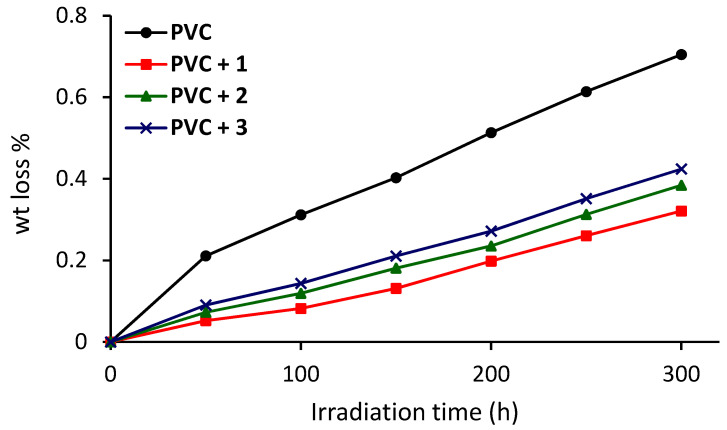
Changes in weight loss (%) of PVC films.

PVC photo-oxidation and photodecomposition lead to cross-linking and bond breaking resulting in the formation of volatiles such as HCl, and the production of small polymeric fragments. The outcome is weight loss by the PVC sample which increases with irradiation time [44]. The damage induced in the PVC chains can be assessed by measuring weight during irradiation and using Equation (2) to calculate the percentage weight loss. The percentage weight losses for the samples as a function of irradiation time (0–300 h) are shown in Figure 4. Relative to the other samples, a sharp weight loss was observed in the first 50 h for the pure PVC sample before settling to a rate fairly similar to the other samples. The weight loss (%) was 0.21% after 50 h and 0.70% after 300 h of irradiation for the pure PVC. The trimethoprim–tin complexes led to a significant drop in the weight loss of PVC. Complex **1** led to the most significant reduction followed by **2** and then by **3**. The blend containing **1** showed a weight loss (%) of 0.05% after 50 h and 0.32% at the end of irradiation. These results are in agreement with those obtained from FTIR and functional group indices.

### 3.4. Investigation of PVC Photodegradation through Surface Morphology

**Figure 5:**
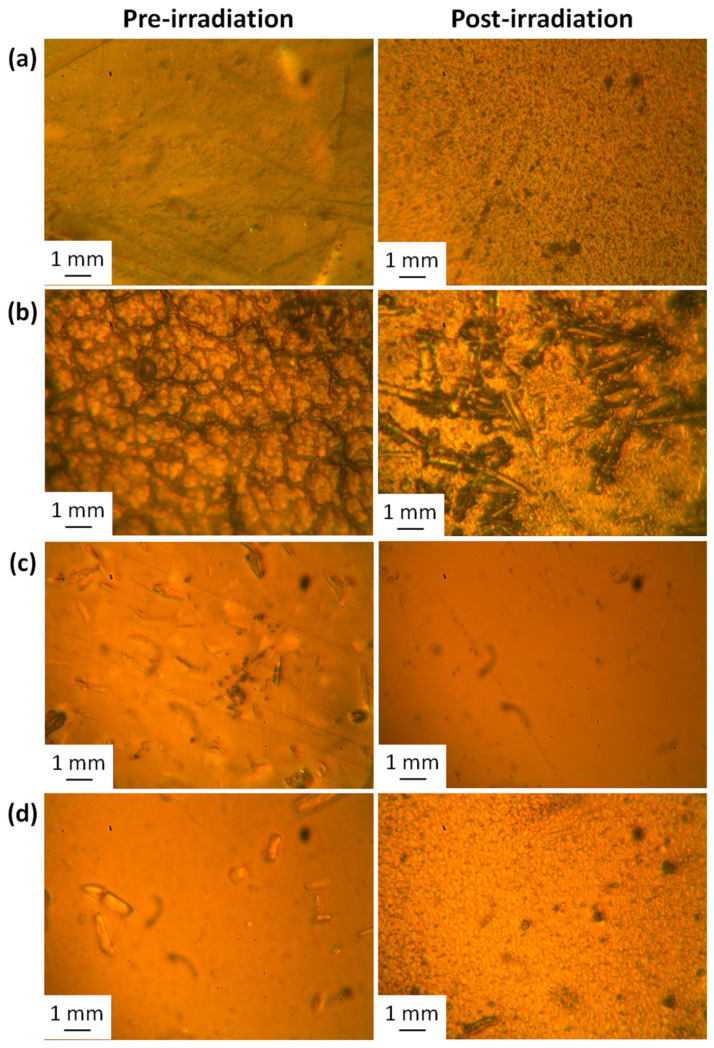
Microscope images of: (**a**) PVC blank (**b**) PVC + **1**, (**c**) PVC + **2**, and (**d**) PVC + **3** blends both before and after irradiation.

Changes on the surface of the PVC film can give an indication of damage during irradiation. With damage, microscope images of the surface of PVC films recorded post-irradiation would show irregularities when compared with pre-irradiation images. The images for the samples are shown in Figure 5. 

In general, the number of cracks, dark spots, and grooves is more noticeable post-irradiation. The appearance of the dark spots is mainly due to the release of HCl because of PVC decomposition [45]. The dark spots were most noticeable on the surface of the pure PVC film. The surface of the blend containing **2** was almost smooth with no groves or crakes. The surface of the blend containing **3** showed some dark spots and irregularities. The surface of the blend containing **1** showed the presence of dark areas which appeared to be rod-shaped. It is not clear why such rodlike shapes were only seen in the case of **1**. However, it could have something to do with the rate of the elimination of volatiles during the photodegradation process. In an attempt to understand the changes on the surface of the PVC blend containing **1**, it was decided to use an additional technique, namely FESEM.

**Figure 6:**
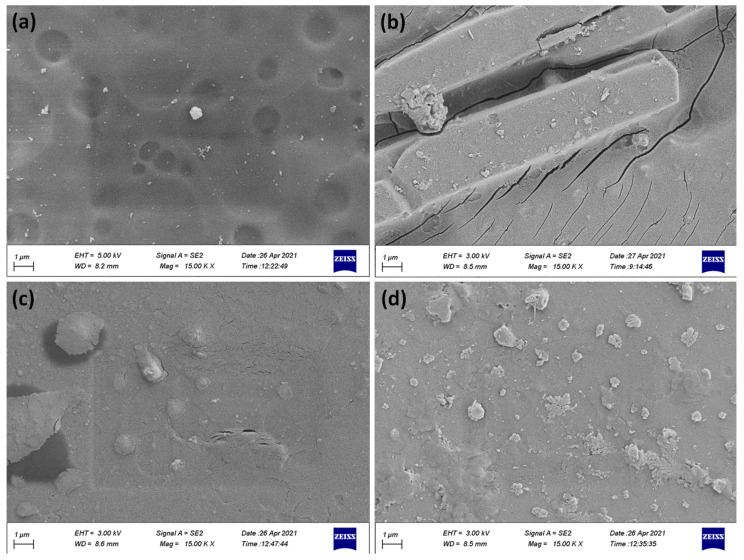
FESEM images of (**a**) PVC, (**b**) PVC + **1**, (**c**) PVC + **2** and (**d**) PVC + **3** films after irradiation.

**Figure 7:**
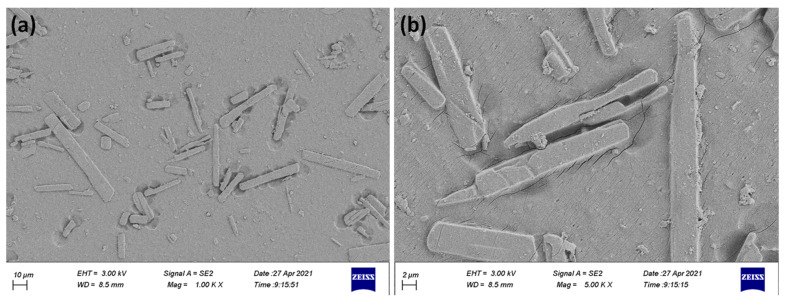
FESEM images of PVC +**1** films after irradiation (**a**) 1× and (**b**) 5× magnification.

The FESEM images gave clearer less distorted and high-resolution pictures of the PVC surface. The images provided information about the homogeneity of the surface as well as the particles size and shape for the irradiated materials [46]. Figure 6 shows that the surface of irradiated PVC films contains lumps, groves, and noticeable irregularities attributed to bond cleavage during photoirradiation and photodegradation [47]. The size of the particles varies; 42.07–132.9 nm for **1**, 69.11–164.8 nm for **2**, and 42.07–152.3 nm for **3**. Again, the surface of the blend containing **1** shows cracks and rodlike particles, which is consistent with the microscope image (Figure 6). It has been reported that PVC containing nickel chloride and a dithiazole Schiff base or tin–naphthalene sulfonic acid complex led to the formation of honeycomb-like structures [31,38]. In addition, PVC blended with a Schiff base containing a melamine unit showed an ice–rock structure [36]. Such observations could be attributed to the slow rate of HCl elimination from the PVC chains during photoirradiation. Figure 7 shows the FESEM images of the particles within the PVC + **1** film post- irradiation at lower magnification.

**Figure 8:**
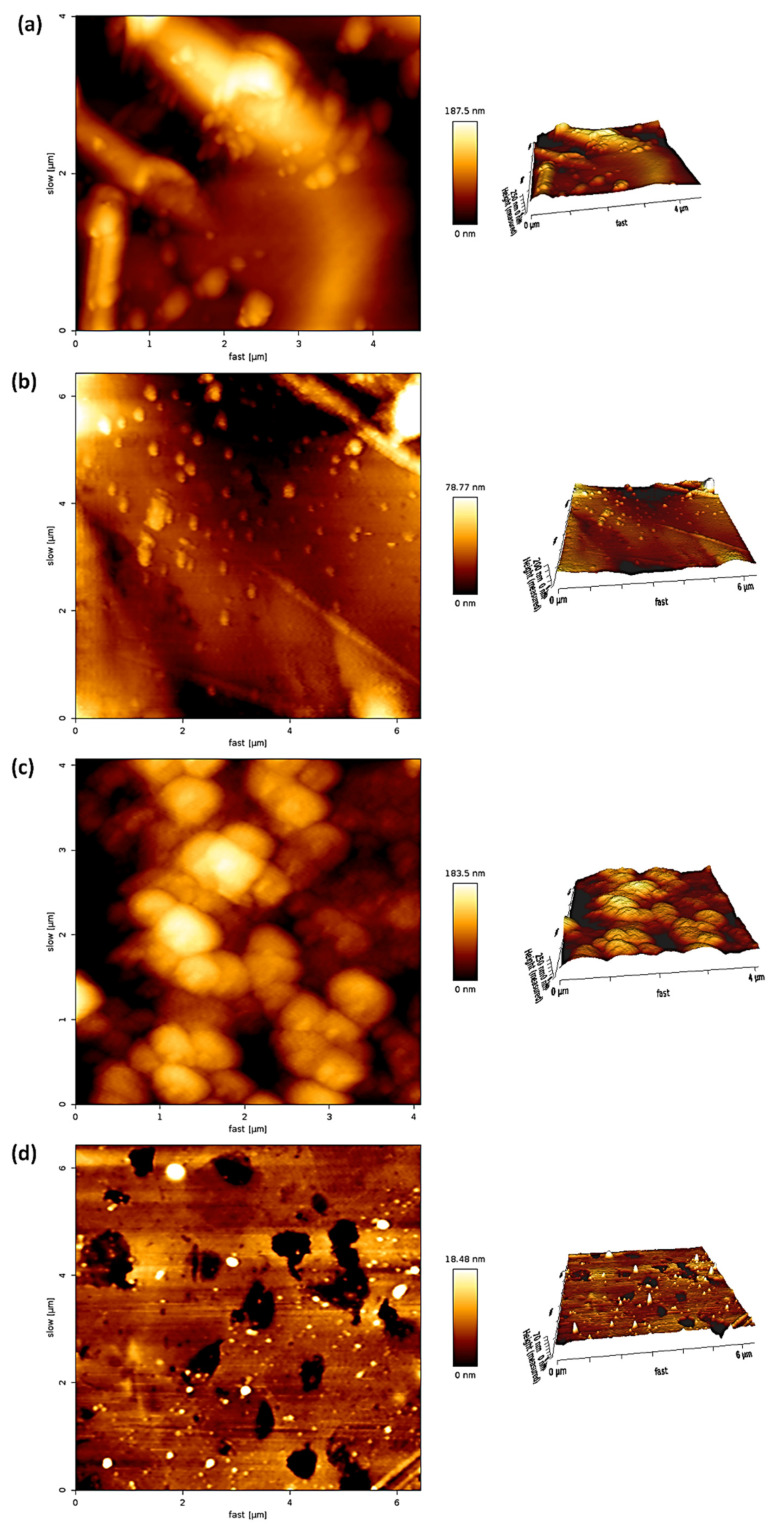
AFM images of (**a**) PVC, (**b**) PVC + **1**, (**c**) PVC + **2** and (**d**) PVC + **3** films.

AFM has also been used to analyze the surface of the irradiated PVC films. Previous reports showed that non-irradiated polymeric films have a high level of homogeneity and smoothness [48,49]. On the other hand, the AFM images of the irradiated blends showed that the surface was badly damaged due to bond cleavage (Figure 8). The surface of the irradiated blends was rough and contained dark spots, particularly in the case of the pure PVC film. Complexes **1–3** reduced the damage caused by photoirradiation, leading to a smoother surface. The roughness factor (*Rq*) for the irradiated pure PVC film was 295.2, which is much higher than for the blends containing the tin complexes. The *Rq* for the PVC blends containing **1**, **2**, and **3** were 26.1, 38.6, and 51.2, respectively. Clearly, the trimethoprim–tin complexes caused a significant reduction in the *Rq* values. For example, complex **1** led to a reduction in the *Rq* by 11.3-fold, which is higher than that achieved with many other organometallic complexes (Table 4).

### 3.5. PVC Photostabilization Mechanisms

**Figure 9:**
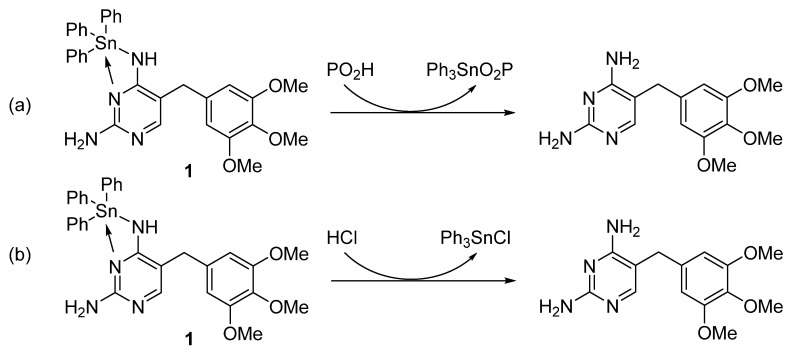
PVC stabilization in the presence of **1** by acting as (**a**) a peroxide decomposer and (**b**) a HCl scavenger.

**Figure 10:**
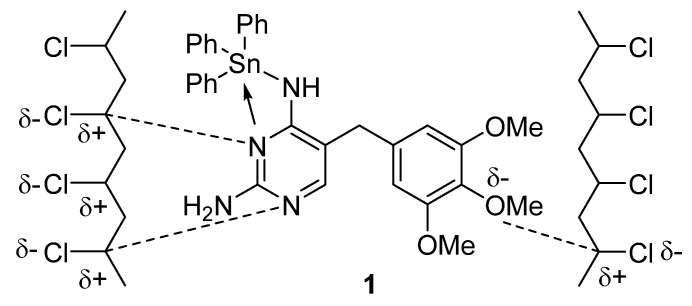
Interaction between complex **1** and PVC.

Trimethoprim–tin complexes **1–3** act as efficient PVC photostabilizers as they are able to absorb UV light. The complexes contain aromatic rings that stabilize the highly energetic intermediates formed on irradiation through resonance [57]. The tin complexes can also decompose hydroperoxide (Figure 9), which is produced during photo-oxidation of PVC [58]. In addition, the tin atom is capable of scavenging the HCl (Figure 9) produced during the irradiation process [52]. Both PVC and tin complexes contain polar bonds (e.g., C–Cl in PVC and C–O and C–N in tin complexes) that allow coordination between them, leading to easier energy transfer from the polymeric chains to the additives (Figure 10). However, complications, such as steric hindrance, that may be associated with large molecules would make such a possibility less likely [53].

## 4. Conclusions

A convenient method for the production of trimethoprim–tin complexes in good yield has been established. The trimethoprim–tin complexes have been shown to act as PVC photostabilizers, leading to a significant reduction in the damage caused by photo-oxidation and photodegradation of the polymeric chains. The additives contain aromatic and heterocyclic moieties that enable them to act as absorbers for ultraviolet light, quenchers for free radicals, and decomposers for hydroperoxides. In addition, the heteroatoms (oxygen and nitrogen) within the trimethoprim–tin complexes facilitate their coordination and interaction with PVC chains. The additives led to a significant decrease in the roughness factor of the PVC surface compared with many other organotin complexes. The additives containing aromatic substituents (phenyl groups) showed better performance as PVC photostabilizers compared with the one containing aliphatic substituents (*n*-butyl groups).

## Figures and Tables

**Table 1:** Physical properties and elemental composition of **1–3**.

Complex	Color	M.P. (°C)	Yield (%)	Elemental Analyses (%) Calculated (Found)			
				C	H	N	Sn
**1**	White	267–269	75	60.12 (60.25)	5.05 (5.10)	8.76 (8.78)	18.57 (18.48)
**2**	Off white	217–218	79	56.42 (56.36)	5.21 (5.25)	13.16 (13.23)	13.94 (14.01)
**3**	White	245–246	82	53.28 (53.27)	6.46 (6.39)	13.81 (13.89)	14.63 (14.76)

**Table 2:** FTIR spectral data for trimethoprim–tin complexes **1–3**.

Complex	FTIR, Wave Number (ν, cm^−1^)				
	NH_2_	NH	C=N	Sn–C	Sn–N
**1**	3406	3176	1680	605	497
**2**	3408	3180	1680	607	586
**3**	3406	3163	1682	605	516

**Table 3:** NMR spectral data of trimethoprim–tin complexes **1–3**.

Complex	^1^H NMR (DMSO-*d_6_*: δ, ppm, *J* in Hz)	^13^C NMR (DMSO-*d_6_*: δ, ppm)
**1**	3.16 (s, exch., 1H, NH), 3.61 (s, 2H, CH_2_), 3.64 (s, 3H, OMe), 3.76 (s, 6H, 2 OMe), 6.64 (s, 2H, 3,4,5-(OMe)_3_C_6_H_2_), 7.47–7.53 (m, 17H, 3 Ph and NH_2_), 7.95 (s, 1H, pyrimidinyl)	32.7 (CH_2_), 56.4 (OMe), 60.4 (OMe), 106.7 (C2/C6 of 3,4,5-(OMe)_3_C_6_H_2_), 109.2 (C5 of pyrimidinyl), 128.5 (C3/C5 of Ph), 128.2 (C4 of Ph), 133.7 (C1 of 3,4,5-(OMe)_3_C_6_H_2_), 136.6 (C4 of 3,4,5-(OMe)_3_C_6_H_2_), 137.0 (C2/C6 of Ph), 137.5 (C1 of Ph), 141.0 (C3/C5 of 3,4,5-(OMe)_3_C_6_H_2_), 153.3 (C4 of pyrimidinyl), 161.1 (C2 of pyrimidinyl), 164.3 (C1 of pyrimidinyl)
**2**	3.46 (s, exch., 2H, 2 NH), 3.56 (s, 4H, 2 CH_2_), 3.64 (s, 6H, 2 OMe), 3.74 (s, 12H, 4 OMe), 6.28 (s, exch., 4H, 2 NH_2_), 6.59 (s, 4H, 3,4,5-(OMe)_3_C_6_H_2_), 7.26–7.36 (10H, 2 Ph), 7.96 (s, 2H, pyrimidinyl)	33.2 (CH_2_), 56.3 (OMe), 60.4 (OMe), 106.4 (C2/C6 of 3,4,5-(OMe)_3_C_6_H_2_), 107.2 (C5 of pyrimidinyl), 127.4 (C3/C5 of Ph), 127.6 (C4 of Ph), 133.1 (C1 of 3,4,5-(OMe)_3_C_6_H_2_), 135.3 (C4 of 3,4,5-(OMe)_3_C_6_H_2_), 135.5 (C2/C6 of Ph), 136.3 (C1 of Ph), 151.5 (C3/C5 of 3,4,5-(OMe)_3_C_6_H_2_), 153.2 (C4 of pyrimidinyl), 160.4 (C2 of pyrimidinyl), 163.2 (C1 of pyrimidinyl)
**3**	0.89 (t, *J* = 7.6 Hz, 6H, 2 Me), 1.30 (sextet, *J* = 7.6 Hz, 4H, 2 MeCH_2_), 1.49 (quintet, *J* = 7.6 Hz, 4H, 2 MeCH_2_CH_2_), 1.69 (t, *J* = 7.6 Hz, 4H, 2 MeCH_2_CH_2_CH_2_), 3.30 (s, exch., 2H, 2 NH), 3.54 (s, 4H, 2 CH_2_), 3.63 (s, 6H, 2 OMe), 3.74 (s, 12H, 4 OMe), 6.11 (s, exch., 4H, 2 NH_2_), 6.58 (s, 4H, 3,4,5-(OMe)_3_C_6_H_2_), 7.51 (s, 2H, pyrimidinyl)	14.2 (Me), 16.5 (SnCH_2_), 26.1 (SnCH_2_CH_2_), 28.1 (Me*C*H_2_), 33.3 (CH_2_), 56.3 (OMe), 60.4 (OMe), 106.4 C2/C6 of 3,4,5-(OMe)_3_C_6_H_2_), 107.0 (C5 of pyrimidinyl), 135.8 (C1 of 3,4,5-(OMe)_3_C_6_H_2_), 136.3 (C4 of 3,4,5-(OMe)_3_C_6_H_2_), 150.0 (C3/C5 of 3,4,5-(OMe)_3_C_6_H_2_), 153.2 (C4 of pyrimidinyl), 161.2 (C2 of pyrimidinyl), 163.1 (C1 of pyrimidinyl)

**Table 4:** Effect of various organotin complexes on the *Rq* for PVC films.

PVC Additive	*Rq* Reduction (Fold)	Reference
Trimethoprim–tin complexes	11.3	[current work]
Naproxen–tin complexes	5.2	[50]
2-(4-Isobutylphenyl)propanoate-nickel complexes	6.3	[51]
Carvedilol–tin complexes	6.4	[36]
Furosemide–tin complexes	6.6	[52]
Valsartan–tin complexes	7.4	[53]
Telmisartan–tin complexes	9.4	[54]
Ciprofloxacin–tin complexes	16.6	[55]
4-(Benzylideneamino)benzenesulfonamide–tin complexes	18.4	[56]

## Data Availability

Data are contained within the article.

## Supplementary Materials

The following are available online at https://www.mdpi.com/article/10.3390/polym13193264/s1, Figure S1: FTIR spectrum of **1**; Figure S2: FTIR spectrum of **2**; Figure S3: FTIR spectrum of **3**; Figure S4: ^1^H NMR spectrum of **3**; and Figure S5: ^13^C NMR spectrum of **3**.
