# Supplementary material for: FTIR, Weight, and Surface Morphology of Poly(vinyl chloride) Doped with Tin Complexes Containing Aromatic and Heterocyclic Moieties
Source: Polymers (Basel). 2021 Sep 25;13(19):3264. doi: 10.3390/polym13193264 (PMC8512238; doi:10.3390/polym13193264)
Supplement: Supplementary file 1 [file polymers-13-03264-s001.zip › polymers-1394114-supplementary.pdf]

polymers

MDPI

Article

FTIR, Weight, and Surface Morphology of Poly(vinyl chloride) Doped with Tin Complexes Containing Aromatic and Heterocyclic Moieties

Anaheed A. Yaseen [1], Emad Yousif [2], Emaad T. B. Al-Tikrity [1], Gamal A. El-Hiti [3,*], Benson M. Kariuki [4], Dina S. Ahmed [5] and Muna Bufaroosha [6]

1 Department of Chemistry, College of Science, Tikrit University, Tikrit 34001, Iraq; ch@sc.nahrainuniv.edu.iq (A.A.Y.); emaad1954@tu.edu.iq (E.T.B.A.-T.)
2 Department of Chemistry, College of Science, Al-Nahrain University, Baghdad 64021, Iraq; emad.yousif@nahrainuniv.edu.iq
3 Department of Optometry, College of Applied Medical Sciences, King Saud University, P.O. Box 10219, Riyadh 11433, Saudi Arabia
4 School of Chemistry, Cardiff University, Main Building, Park Place, Cardiff CF10 3AT, UK; kariukib@cardiff.ac.uk
5 Department of Medical Instrumentation Engineering, Al-Mansour University College, Baghdad 64021, Iraq; dina.saadi@muc.edu.iq
6 Department of Chemistry, College of Science, United Arab Emirates University, P.O. Box 15551, Al-Ain 1818, United Arab Emirates; muna.bufaroosha@uaeu.ac.ae
* Correspondence: gelhiti@ksu.edu.sa; Tel.: +966-11469-3778; Fax: +966-11469-3536

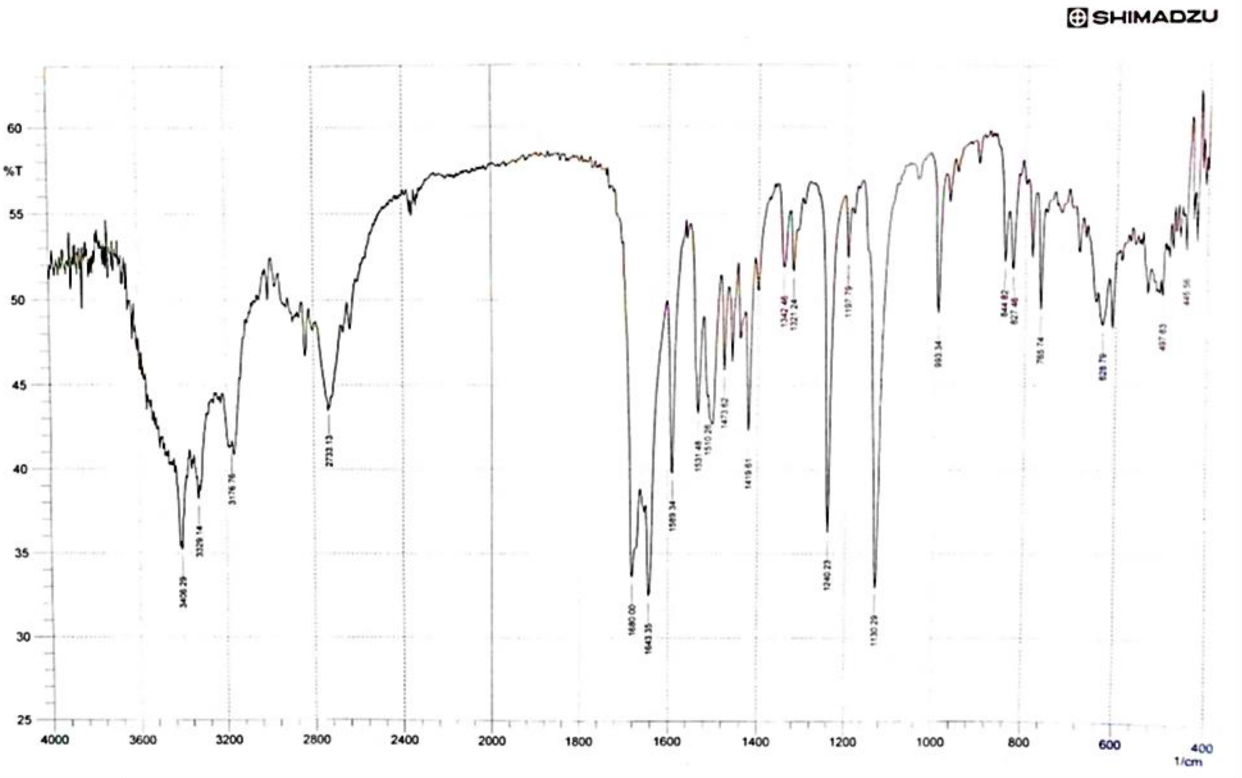

Figure S1. FTIR spectrum of **1**.

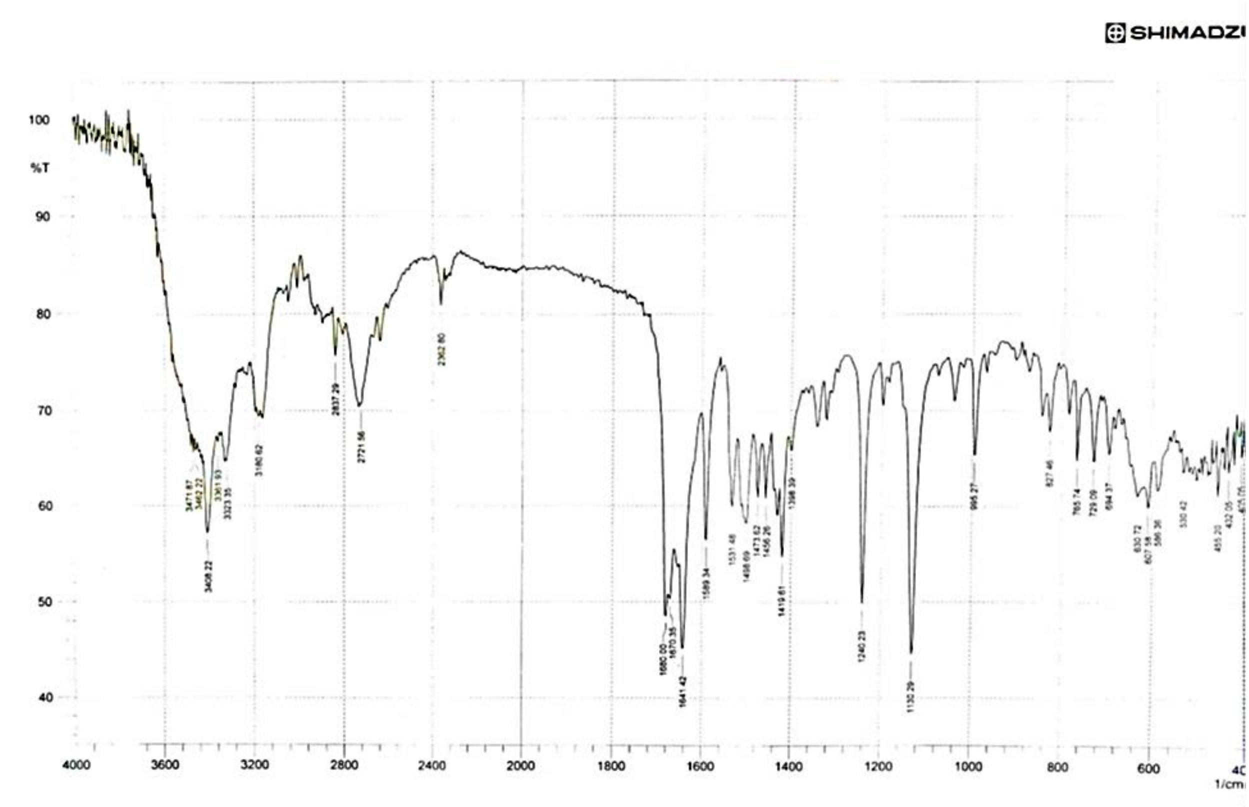

Figure S2. FTIR spectrum of **2**.

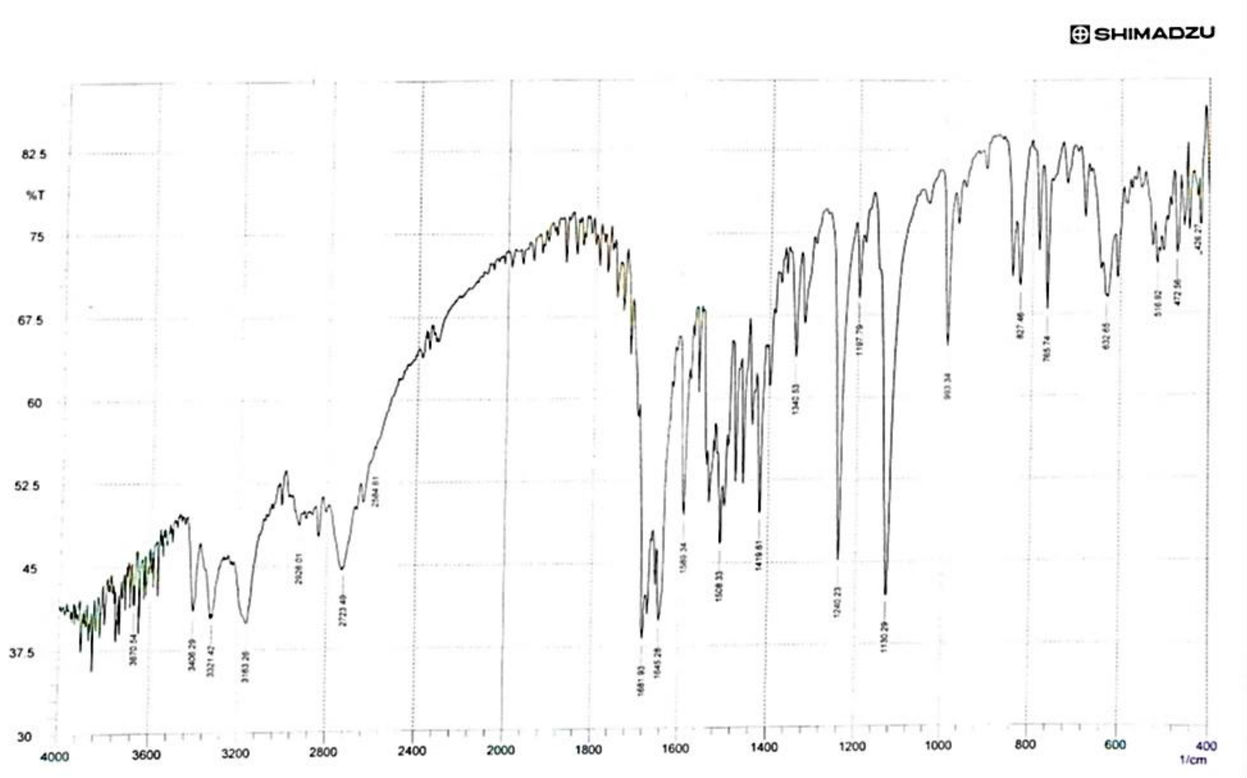

Figure S3. FTIR spectrum of **3**.

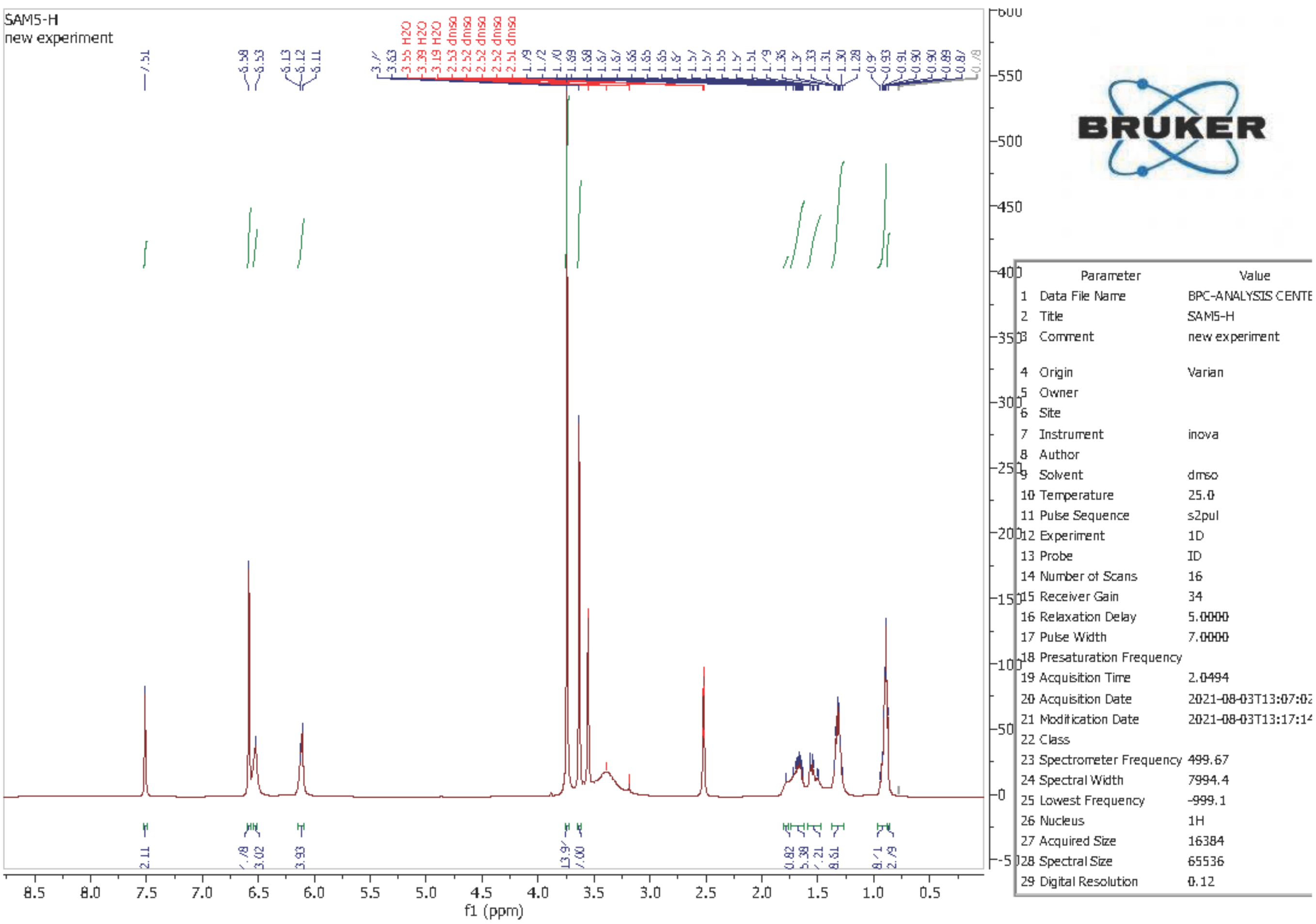

Figure S4. ^{1}H NMR spectrum of **3**.

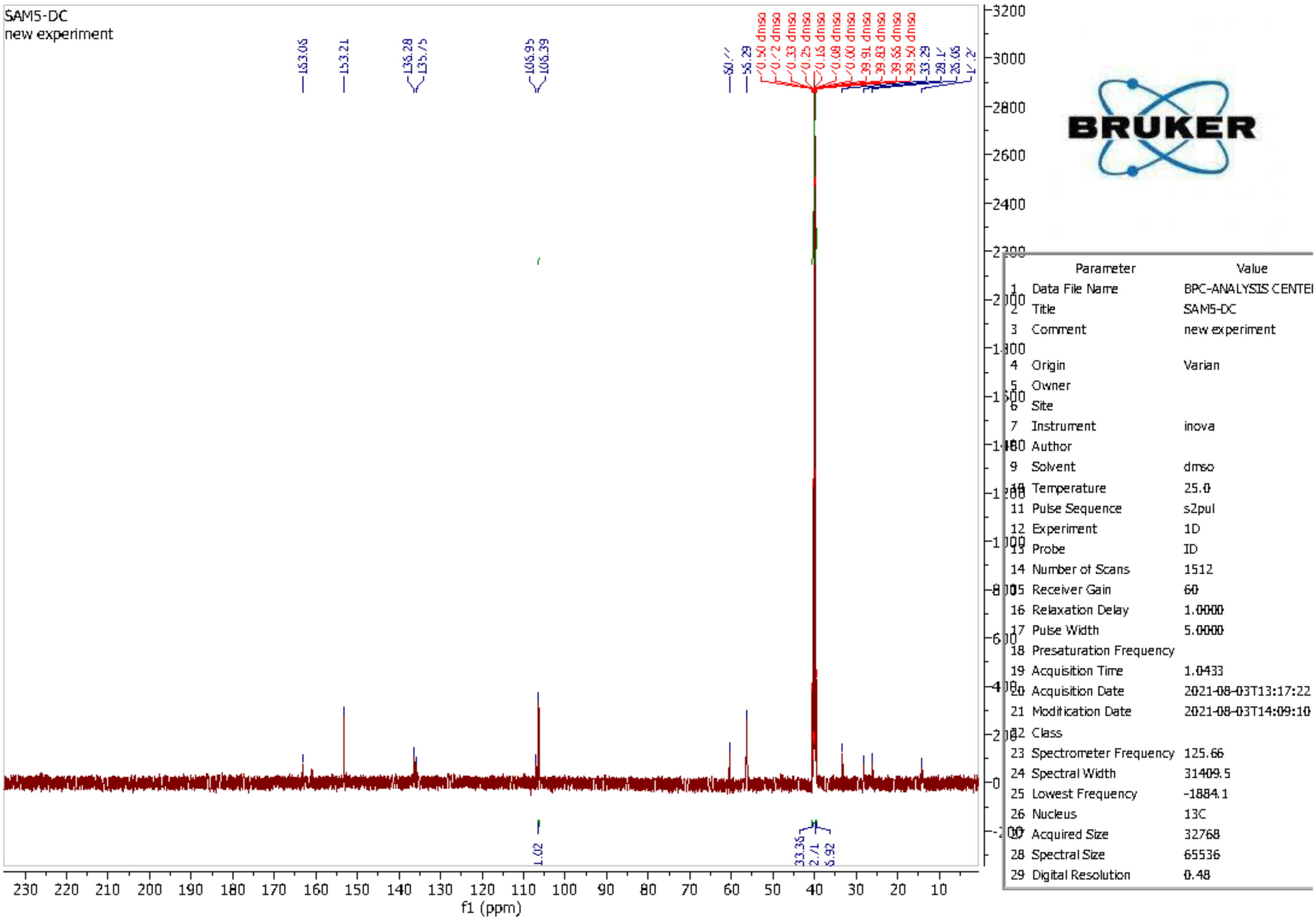

Figure S5. ^{13}C NMR spectrum of **3**.
